# A Simple, Secure and Universal Pancreaticojejunostomy following Pancreaticoduodenectomy

**DOI:** 10.1155/1997/10729

**Published:** 1997

**Authors:** P.-W. Lin, J.-C. Lee, P.-C. Lee, T.-W. Chang, C.-J. Hung, Y.-C. Chang

**Affiliations:** Department of Surgery Medical College National Cheng Kung University 138 Sheng-Li Road Tainan Taiwan

## Abstract

Although the operative mortality of pancreaticoduodenal resection has decreased recently, the operative morbidity resulting from a leaking pancreatic anastomosis remains high. We described our experience in 50 consecutive cases with a simple, secure end to side pancreaticojejunostomy. We used a paediatric nasogastric tube in the pancreatic remnant duct as a temporary external pancreatic drain. There were 29 men and 21 women ranging from 12 to 84 years with a median age of 61 years. Forty-two patients underwent a standard Whipple procedure and eight a pylorus preserving pancreaticoduodenectomy. Average operating time was 270 minutes with a range of 170 to 480 minutes. The pancreaticojejunostomy could be constructed in a mean of 8 minutes. Intraoperative blood loss ranged from 150 to 3500 mL with a mean of 910mL. Twenty-five patients (50 %) received no blood transfusion. The consistency of the pancreatic remnant was hard in 12 patients (24 %) and normal in 38 patients (76 %). The pancreatic duct was dilated (>4mm) in 15 patients (30 %). There was no operative mortality and only three (6.0 %) minor leaks from the pancreatic anastomosis which healed spontaneously. It was difficult to determine if the leaks were related to the consistency of the pancreatic remnant, the size of the pancreatic duct, the amount of intraoperative blood loss, operating time, sex of the patient or experience of the surgeon, as there were only three leaks. We concluded that our technique for pancreaticojejunal anastomosis following pancreaticoduodenectomy was safe and applicable to, standard Whipple or pylorus preserving pancreaticoduodenectomy, small or dilated pancreatic ducts, normal or fibrotic pancreas.


*HPB Surgery*, 1997, Vol. 10, pp. 305-310
Reprints available directly from the publisher
Photocopying permitted by license only

© 1997 OPA (Overseas Publishers Association)
Amsterdam B.V. Published in The Netherlands
by Harwood Academic Publishers
Printed in India

### A Simple, Secure and Universal Pancreaticojejunostomy following Pancreaticoduodenectomy

P.-W. LIN*, J.-C. LEE, P.-C. LEE, T.-W. CHANG, C.-J. HUNG and Y.-C. CHANG

*Department of Surgery, Medical College, National Cheng Kung University, 138 Sheng-Li Road, Tainan, Taiwan*

*(Received 1 June 1996; In final form 1 October 1996)*

**Although the operative mortality of pancreaticoduodenal resection has decreased recently, the operative morbidity resulting from a leaking pancreatic anastomosis remains high. We described our experience in 50 consecutive cases with a simple, secure end to side pancreaticojejunostomy. We used a paediatric nasogastric tube in the pancreatic remnant duct as a temporary external pancreatic drain. There were 29 men and 21 women ranging from 12 to 84 years with a median age of 61 years. Forty-two patients underwent a standard Whipple procedure and eight a pylorus preserving pancreaticoduodenectomy. Average operating time was 270 minutes with a range of 170 to 480 minutes. The pancreaticojejunostomy could be constructed in a mean of 8 minutes. Intraoperative blood loss ranged from 150 to 3500 mL with a mean of 910mL. Twenty-five patients (50 %) received no blood transfusion. The consistency of the pancreatic remnant was hard in 12 patients (24 %) and normal in 38 patients (76 %). The pancreatic duct was dilated (>4 mm) in 15 patients (30 %). There was no operative mortality and only three (6.0 %) minor leaks from the pancreatic anastomosis which healed spontaneously. It was difficult to determine if the leaks were related to the consistency of the pancreatic remnant, the size of the pancreatic duct, the amount of intraoperative blood loss, operating time, sex of the patient or experience of the surgeon, as there were only three leaks. We concluded that our technique for pancreaticojejunal anastomosis following pancreaticoduodenectomy was safe and applicable to, standard Whipple or pylorus preserving pancreaticoduodenectomy, small or dilated pancreatic ducts, normal or fibrotic pancreas.**

*Keywords:* Pancreaticoduodenectomy, pancreaticojejunostomy, anastomotic leak

### INTRODUCTION

Although the operative mortality of pancreaticoduodenal resection has improved recently, the operative morbidity resulting from a leaking pancreatic anastomosis remains high [1–12]. A variety of approaches to the management of the pancreatic remnant and duct have been tried [13–22]. Even in large series, a substantial leak rate from the pancreatic anastomois has been reported [3–12]. Some leaks require reoperation, either widespread peripancreatic drainage with/without anastomotic repair or completion pancreatectomy, and may lead to operative

*Author for correspondence.

mortality [7, 8, 23]. Herein, we report our experience with 50 consecutive pancreaticoduodenal resections without any operative mortality and with a low rate (6.0 %) of minor leak from the pancreaticojejunal anastomosis. A simple, universal, but very secure end-to-side pancreticojejunostomy was used in all patients with an appropriate paediatric nasogastric tube in the pancreatic remnant duct as a temporary external pancreatic drain for 3 weeks.

### PATIENTS AND METHODS

From April 1989 to June 1995, 50 consecutive patients underwent pancreaticoduodenectomy at National Cheng Kung University Hospital for a variety of indications. (Tab. I) There were 29 men and 21 women with a median age of 61 years (range 12 to 84). Eleven patients were 70 years of age or older, and two were over 80. The youngest patient was a 12-year-old girl, who had a large papillary cystic neoplasm at the head of the pancreas. A classic pancreaticoduodenectomy (with subtotal gastrectomy) was performed in 42 patients (84 %), and a pylorus preserving pancreaticoduodenectomy (PPPD) in eight patients (16 %); five patients had a concomitant right hemicolectomy due to suspicious malignant invasion to the mesocolon and/or the colon (three pancreatic cancer, one ampulla vater cancer and one gastric cancer); one had a partial resection of the inferior vena cava with direct repair; one had a concomitant classic Whipple and right hepatic lobectomy. (The patient had a previous left hemicolectomy for colon cancer 12 years ago and a right nephroureterectomy for transitional cell carcinoma of the renal pelvis three years ago. During this hospitalization, he had a biopsy proven ampulla of vater cancer with an isolated hepatic mass in segment 7. The resected liver specimen revealed chronic cholangitis with periductal fibrosis and lymphocytic infiltraltion. The postoperative course was uneventful.) Only one type (described below) of pancreaticojejunal anastomosis, a modification of the method used by Smith [13], was used. Ninety percent of the cases were performed by our senior author (P.W.L.). Somatostatin was not used in any of these patients.

### Technique for Pancreaticojejunostomy

After the pancreaticoduodenal resection was completed, the proximal end of the jejunum was closed with staples. The proximal jejunal loop was brought antecolically to the cut end of the common hepatic duct. A single layer anastomosis in an end to side fashion between the common hepatic duct and the jejunum with interrupted 3-O silk sutures but without a T-tube was performed. The pancreaticojejunal anastomosis was then reconstructed. An appropriate paediatric nasogastric tube, according to the diameter of the pancreatic remnant duct, was inserted into the pacreatic duct, and secured with a suture of 3-O chromic catgut. The nasogastric tube was pulled through the jejunum via a stab wound in both sides of the jejunum, which was 10 to 15 cm distal to the hepaticojejunostomy, and was brought out extraabdominally. A row of 3-O silk sutures were placed through the posterior pancreatic parenchyma, the pancreatic capsule and the posterior seromuscular layer of the jejunum. Then, these sutures were tied to approximate the pancreatic remnant and the jejunum posteriorly. (Fig. 1) The anastomosis was completed with several interrupted 3-O silk sutures between the anterior

TABLE I Indications for 50 pancreaticoduodenectomies

| | |
|---|---|
| Ampulla Vater cancer | 28(1) |
| Pancreatic cancer | 10 |
| Distal CBD cancer | 3 |
| Duodenal leiomyoma | 1 |
| Pancreatic serous cystadenoma | 1(1) |
| Papillary cystic neoplasm | 1 |
| Gastric cancer | 3 |
| Ectopic pancreas of distal CBD | 1 |
| Chronic pancreatitis | 2(1) |
| Total | 50(3)(6.0%) |

( ):Number of Patients with leak.

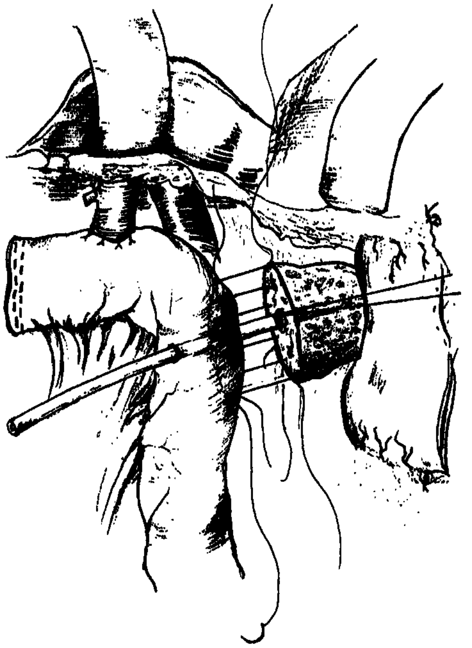

FIGURE 1 Posterior pancreaticojejunal anastomosis was performed by approximating the seromuscular posterior portion of the jejunum to the pancreas with 3-0 silk sutures after the paediatric nasogastric tube was inserted and secured in the pancreatic duct with a suture of 3-0 chromic catgut.

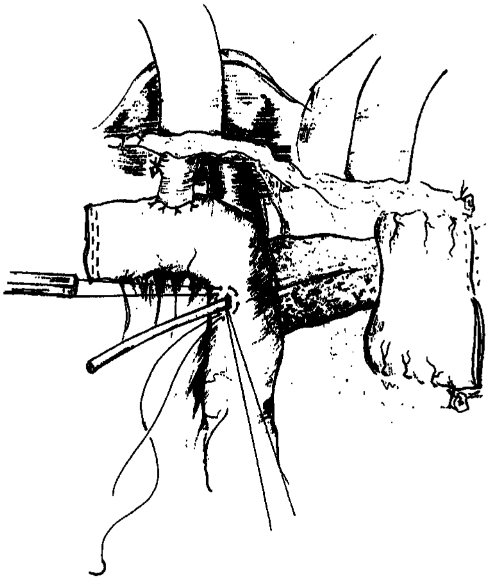

FIGURE 2 The pancreaticojejunal anastomosis was completed after approximating the seromuscular anterior portion of the jejunum to the pancreas. The stenting tube was secured at the exit with a suture of chromic catgut. The exit was strengthened with a pursestring suture of 3-0 silk.

pancreatic parenchyma, the pancreatic capsule and the anterior seromuscular layer of the jejunum. (Fig. 2) The paediatric nasogastric tube was secured with a 3-O chromic catgut suture which was placed near its exit in the jejunum. Finally, a pursestring suture in the seromuscular layer of the jejunum with 3-O silk was used to strengthen the exit. Completion of the pancreaticoduodenectomy and reconstruction was shown in Figure 3. The stenting pancreatic duct tube was removed on the 21st day after operation.

No special preparation of the cut end of the pancreas or the pancreatic duct was needed. No direct mucosa to mucosa anastomosis between the pancreatic duct and the jejunum was performed whether the main pancreatic duct was dilated/normal or the pancreatic remnant was fibrotic/normal. A Jackson-Pratt drain was

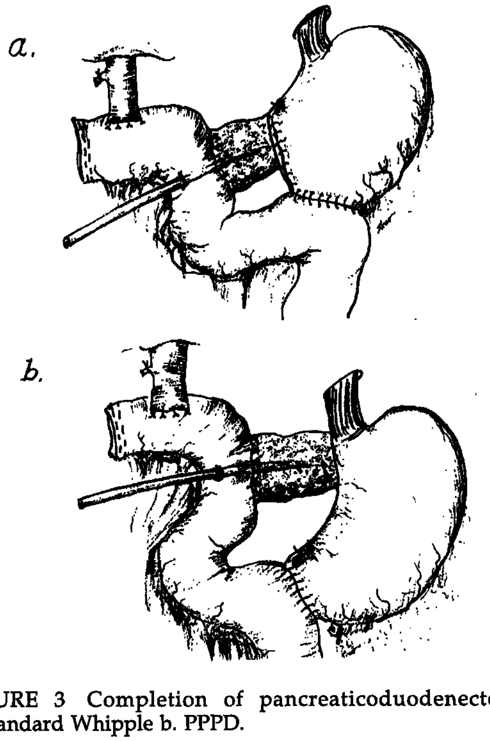

FIGURE 3 Completion of pancreaticoduodenectomy a. Standard Whipple b. PPPD.

placed along the subhepatic space through the foramen Winslow to the lesser sac area.

### RESULTS

There was no operative mortality. Mean duration of operation was 270 minutes (range 170 to 480 minutes). All operations took less than 6 hours except one, in whom a large papillary cystic neoplasm of the pancreas at the head compressing the portal vein and superior mesenteric vein was resected. Pancreaticojejunostomy averaged 8 minutes with a range from 7 to 9 minutes. Intraoperative blood loss ranged from 150 to 3500 mL with a mean of 910 mL. Twenty-five patients (50 %) received no blood transfusion intraoperatively. Pancreatic consistency was hard in 12 patients (24 %) and normal in 38 patients (76 %). The diameter of the pancreatic duct of the remnant ranged from 1 mm to 14 mm with a mean diameter of 4.5 mm. The pancreatic duct was dilated (greater than 4 mm) in 15 patients (30 %). The paediatric nasogastric tube used for external pancreatic duct stenting ranged from Fr5 to Fr14. The daily amount of pancreatic juice collected from the stenting tube ranged from 0 to 1530 mL. A great variation in the daily amount of pancreatic drainage was noted, even in the same patient.

No leakage from the hepaticojejunostmy or gastrojejunostomy was noted. Mild leakage of the pancreaticojejunostomy was noted in three patients, which healed spontaneously in 1 to 3 weeks after conservative treatment. All these three leaks occurred in patients operated on by the senior surgeon. (Tab. II) Mild abdominal pain and increased amylase-rich discharge varying from 50–300 mL daily from the Jackson-Pratt drain occurred in these patients. Because there were only three leaks in this series, it was difficult to comment on any predisposing factors.

### DISCUSSION

A variety of approaches to the management of the pancreatic remnant and duct following pancreaticoduodenectomy have been tried [13–22]. But they were technically complicated [13–22] and the leakage rate remained high [14–22]. Our technique, a modification of the method used by Smith [13], for pancreaticojejunostmy and hepaticojejunostomy was simple. No special preparation for the cut surface of the pancreatic remnant and duct was required as in the dunking procedure or duct to mucosa anastomosis. The anastomosis could be completed in 7 to 9 minutes, and could be universally applied to any situation no matter what the diameter of the pancreatic duct was or what the consistency of the remnant was.

In our pancreaticojejunostomy, we used a pancreatic stent to temporarily drain the pancreatic juice extraabdominally for 3 weeks. No pancreatic juice entered the alimentary tract in the postoperative period. Most importantly, there was only a very small stab wound in the jejunum for the pancreaticojejunal anastomosis. In the three leaks, the volume of drainage varied

TABLE II Three pancreaticojejunal anastomotic leaks

| Sex | Age | diagnosis | Operation time (min) | blood loss (mL) | remnant | Pancreatic duct (mm) |
|---|---|---|---|---|---|---|
| M | 71 | Pancreatic serous adenoma | 260 | 700 | soft | 4 |
| M | 44 | chronic pancreatitis | 267 | 850 | hard | 5 |
| F | 43 | ampulla vater cancer | 275 | 1200 | soft | 1 |

from 50 to 300 mL daily. Only pancreatic juice, almost no biliary-enteric content, was noted from the intraoperatively placed drain tubes. These leaks healed spontaneously with conservative treatment. We did not use somatostatin in any of the patients. Though a large portion of pancreatic leaks can be managed with conservative measures, some leaks require reoperation with either widespread peripancreatic drainage with or without anastomotic repair or completion pancreatectomy. Leaks and reoperation carry a risk of major morbidity, even death [7, 8, 23]. Fortunately, we did not experience severe complications from the anastomotic leaks.

Factors previously reported as influencing the development of a pancreatic leak include a small pancreatic duct, soft or normal pancreatic parenchyma, large intraoperative blood loss, and age over 65 years [2–4]. In this study, only three minor leaks were noted. We were unable to identify any preoperative or operative parameters predisposing to pancreatic anastomotic leaks. However, a delicate surgical technique, we believe, is essential and important to achieve zero mortality and zero anastomotic leak in pancreaticoduodenectomy.

We concluded that our technique for pancreaticojejunostomy was simple, safe and universally applicable to any situation, standard Whipple or PPPD, small or dilated pancreatic ducts, normal or fibrotic pancreatic remnant.

### *Acknowledgment*

The authors would like to thank Prof Shin-Tai Wang for his statistical analysis and Miss Hsueh-Hua Hou for her preparing the manuscript.

### *References*

[1] Howard, J. M. (1968). Pancreatico-duodenectomy: Forty-one consecutive Whipple resections without an operative mortality. *Annals of Surgery*, **168**, 629–640.

[2] Gilsdorf, R. B. and Spanos, P. (1973). Factors influencing morbidity and mortality in pancreatic-oduodenectomy. *Annals of Surgery*, **177**, 332–337.

[3] Lerut, J. P., Gianello, P. R., Otte, J. B. and Kestens, D. J. (1984). Pancreaticoduodenal resection. Surgical experience and evaluation of risk factors in 103 patients. *Annals of Surgery*, **199**, 432–437.

[4] Grace, P. A., Pitt, H. A., Tompkins, R. K., DenBesten, L. and Longmire, W. P. (1986). Decreased morbidity and mortality after pancreatoduodenectomy. *American Journal of Surgery*, **151**, 141–149.

[5] Crist, D. W., Sitzmann, J. V. and Cameron, J. L. (1987). Improved hospital morbidity, mortality and survival after the Whipple procedure. *Annals of Surgery*, **206**, 358–365.

[6] Trede, M. Schwall, G. and Saeger, H. D. (1990). Survival after pancreatoduodenectomy. 118 consecutive resections without an operative mortality. *Annals of Surgery*, **211**, 447–458.

[7] Cullen, J. J., Sarr, M. G. and Ilstrup, D. M. (1994). Pancreatic anastomotic leak after pancreaticoduodenectomy: Incidence, significance, and management. *American Journal of Surgery*, **168**, 295–298.

[8] Miedema, B. W., Sarr, M. G., Van Heerden, J. A., Nagorney, D. M., McIlrath, D. C. and Ilstrup, D. (1992). Complications following pancreaticoduodenectomy. Current management. *Archives of Surgery*, **127**, 945–950.

[9] Braasch, J. W., Deziel, D. J., Rossi, R. L., Watkins, E. and Winter, P. F. (1986). Pyloric and gastric preserving after pancreatic resection. Experience with 87 patients. *Annals of Surgery*, **204**, 411–418.

[10] Cameron, J. L., Pitt, H. A., Yeo, C. T., Lillemoe, K. D., Kaufman, H. S. and Coleman, J. (1993). One hundred and forty-five consecutive pancreaticoduodenectomy without mortality. *Annals of Surgery*, **217**, 430–438.

[11] Pellegrini, C. A., Heck, C. F., Raper, S. and Way, L. W. (1989). An analysis of the reduced morbidity and mortality rates after pancreaticoduodenectomy. *Archives of Surgery*, **124**, 778–787.

[12] Watanapa, P. and Williamson, R. C. N. (1995). Resection of the pancreatic head with or without gastrectomy. *World Journal of Surgery*, **19**, 403–408.

[13] Smith, R. (1973). Progress in the surgical treatment of pancreatic disease. *The American Journal of Surgery*, **125**, 143–153.

[14] ReMine, W. H., Priestley, J. T., Judd, E. S. and King, J. H. (1970). Total pancreatectomy. *Annals of Surgery*, **172**, 595–604.

[15] Cooperman, A. M., Herter, F. P., Marboe, C. A., Helmreich, Z. V. and Perzin, K. H. (1981). Pancreatoduodenal resection and total pancreatectomy-an institutional review. *Surgery*, **90**, 707—712.

[16] Mason, G. R. and Freeark, R. J. (1995). Current experience with pancreatogastrostomy. *American Journal of Surgery*, **169**, 217–219.

[17] Goldsmith, H. S., Ghosh, B. C. and Huvos, A. G. (1971). Ligation versus implication of the pancreatic duct after pancreaticoduodenectomy. *Surgery Gynencology Obstetrics*, **32**, 87–92.

[18] Tashiro, S., Murata, E., Hiraoka, H., Nakakuma, K., Watanabe, E. and Miyauchi, Y. (1987). New technique for pancreaticojejunostomy using biological adhesive. *British journal of Surgery*, **74**, 392–394.

[19] Hiraoka,T., Kanemitsu, K. and Tsuji, T. *et al.* (1993). A method for safe pancreaticojejunostomy. *American Journal of Surgery*, **165**, 270–272.

[20] Manabe, T., Suzuki, T. and Tobe, T. (1986). A secured technique for pancreatojejunal anastomosis in pancreaticoduodenectomy. *Surgery Gynencology Obstetrics*, **163**, 379–380.

[21] Matsumoto, Y., Fujii, H. and Miura, K. *et al.* (1992). Successful pancreatojejunal anastomosis for pancreatoduodenectomy. *Surgery Gynencology Obstetrics*, **175**, 555–562.

[22] Lygidakis, N. J., Savanis, G., Touloupakis, T. and Pothoulakis, I. (1993). Technical considerations and results of a "New" method of reconstruction of alimentary continuity after duodenopancreatectomy. *Hepato-gastroenterology*, **40**, 448–451.

[23] Smith, C. D., Sarr, M. G. and Van Heerden, J. A. (1992). Completion pancreatectomy following pancreatico-duodenectomy: clinical experience. *World Journal of Surgery*, **16**, 521–524.

